# Potential for Development of an *Escherichia coli*—Based Biosensor for Assessing Bioavailable Methionine: A Review

**DOI:** 10.3390/s100403562

**Published:** 2010-04-08

**Authors:** Vesela I. Chalova, Clifford A. Froelich, Steven C. Ricke

**Affiliations:** 1 Poultry Science Department, Texas A&M University, College Station, TX 77843-2472, USA; E-Mails: veselachalova@gmail.com (V.I.C.); CFroel@lsuhsc.edu (C.A.F.); 2 Center for Food Safety and Department of Food Science, University of Arkansas, Fayetteville, AR 72704, USA

**Keywords:** methionine, microbial biosensors, *Escherichia coli*, bioavailability

## Abstract

Methionine is an essential amino acid for animals and is typically considered one of the first limiting amino acids in animal feed formulations. Methionine deficiency or excess in animal diets can lead to sub-optimal animal performance and increased environmental pollution, which necessitates its accurate quantification and proper dosage in animal rations. Animal bioassays are the current industry standard to quantify methionine bioavailability. However, animal-based assays are not only time consuming, but expensive and are becoming more scrutinized by governmental regulations. In addition, a variety of artifacts can hinder the variability and time efficacy of these assays. Microbiological assays, which are based on a microbial response to external supplementation of a particular nutrient such as methionine, appear to be attractive potential alternatives to the already established standards. They are rapid and inexpensive *in vitro* assays which are characterized with relatively accurate and consistent estimation of digestible methionine in feeds and feed ingredients. The current review discusses the potential to develop *Escherichia coli*-based microbial biosensors for methionine bioavailability quantification. Methionine biosynthesis and regulation pathways are overviewed in relation to genetic manipulation required for the generation of a respective methionine auxotroph that could be practical for a routine bioassay. A prospective utilization of *Escherichia coli* methionine biosensor would allow for inexpensive and rapid methionine quantification and ultimately enable timely assessment of nutritional profiles of feedstuffs.

## 1. Introduction

Methionine is an essential amino acid for animals and is involved in numerous metabolic processes [1–4]. In addition to being a building block in protein synthesis, methionine, after being transformed into S-adenosylmethionine, serves as a methyl donor in transmethylation reactions involved in the biosynthesis of lipids, biotin, and polyamines [5]. Since methionine cannot be synthesized *de novo* in mammal cells, its supplementation in animal diets is required to provide optimal growth and physiological performance of the animals. Plant proteins, however, are poor in methionine and its optimal level in animal diets is provided by supplementation with crystalline methionine [6] or methionine analogs such as 2-keto-4-(methylthio) butyric acid [7] and hydroxymethionine [8,9]. Therefore, timely and accurate pre-quantification of this amino acid in feed ingredients is necessary to improve cost efficiency of feed formulation and prevent its overdosage. According to Klasing and Austic [10] and Baker [11], excess of individual amino acids due to feed mixing errors can be potentially harmful to the animal, with methionine considered to be the amino acid possessing the highest toxicity. Feed compounds such as cysteine, vitamin B_12_, arginine, choline, and sulfate that are related to methionine metabolism can affect the apparent methionine requirement of animals and additionally complicate the estimation of the optimal dosage of this amino acid in animal diets [12].

Chemical assays including high performance liquid chromatography (HPLC) are commonly used to quantify methionine level in feed ingredients. The analysis, however, involves pretreatment of the samples with performic acid followed by acid digestion [13,14]. The procedure results in a complete protein degradation and liberation of methionine which differs from protein digestion under physiological conditions. Feed-derived methionine, which is available to animals to assimilate, can be more accurately estimated by animal or microbial assays which are considered to correspond more directly to the physiological needs of animals [15,16]. Although considered standard, animal assays are laborious, expensive, and time consuming [17–19]. The types of animal assays that have been used for quantifying methionine availability have been reviewed extensively by Froelich and Ricke [18] and will not be discussed further in the current review. Microbial assays appear to be easier and more affordable for routine analysis. Rapid development and recent improvements in molecular techniques allow for constructing successful and accurate amino acid biosensors via more precise genetic targeting of specific genes in microbial cells. This review discusses methionine biosynthesis and regulation in *Escherichia coli* and the potential of genetically modifying this microorganism into practical whole cell biosensors for methionine bioavailability quantification.

## 2. Microbial Biosensors

Recently, numerous microbial biosensors have been created and used in medical diagnostics, food technology, biotechnology, and environmental monitoring. Microbial biosensors couple a biological element (enzymes, viable or non-viable microbial cells) and a transducer or a device which allows for rapid, accurate and sensitive detection of target analytes [20,21]. Their popularity is due to highly specific selectivity to the substrate of interest, relative inexpensiveness, and portability [22,23]. Versatile microorganisms have proven to be useful in development of biosensors. The bacterium *Vibrio harveyi* and *Mycena citricolor*, a fungal microorganism, demonstrated high sensitivity for detecting cyanide and sodium monofluoroacetate respectively [24]. A microbial biosensor for sensitive, selective, rapid, and direct determination of *p*-nitrophenyl (PNP) -substituted organophosphates was developed based on PNP oxidation metabolic pathway of the *Moraxella* sp. [25]. *Flavobacterium* sp. were employed for development of a biosensor for methyl parathion pesticide [26]. The variety and versatility among microbial species useful in the construction of biosensors for environmental application is more extensively discussed elsewhere [20,27] and will not be further discussed here.

In the food industry, microbial biosensors, derived from *Gluconobacter oxydans* and yeast have gained popularity for detecting total sugars, sucrose, and ethanol [28,29]. Respiratory activities of *Gluconobacter oxydans* DSM 2343 cells, immobilized on chitosan, were used in the quantification of glucose. A linear relationship (R^2^ = 0.99) between sensor’s response and substrate concentration was achieved in the range of 0.05 to 0.1 mM glucose [23]. By using a microbial biosensor based on immobilized *Saccharomyces ellipsoideus* yeast cells, Rotariu *et al*. [29] were able to determine ethanol concentrations up to 50 mM in alcoholic beverages including two types of beer, vodka, and cognac. The comparison to the chemical assay used for the analyses of the same analyte revealed good correlation (correlation coefficient 0.998) between the biosensor and the spectrometric method. An *Acetobacter pasteurianus*-based biosensor has been proposed as an alternative to chemical methods available for quantifying lactate which is used as an indicator for specific fermentations activities including those of milk, yogurt, and wine [30,31]. *Aeromonas phenologenes*-, *Pseudomonas fluorescens*-, and *Bacillus subtilis*-based biosensors were proposed to serve as alternatives in quantification of amino acids including tyrosine, tryptophan, and glutamate [32,33].

## 3. *E. coli* as a Biosensor

Among all microorganisms, *E. coli* is one of the most highly investigated bacteria for the purposes of biosensor fabrication. It is easy to cultivate, with simple nutritive requirements and rapid growth [34]. *E. coli* is a Gram negative microorganism with very well known genetics which enables the construction of a wide variety of biosensors [20,21]. The complete *E. coli* genome has been sequenced and the information deposited to the National Center for Biotechnology Information (NCBI) [35]. Thus, each DNA sequence of interest is routinely available to the public and can be used for a wide range of potential further genetic manipulations. In promoter-based *E. coli* biosensors, a gene promoter, inducible by the analyte of interest, is fused to a reporter that generates a signal in response to the analyte that can be easily monitored and measured. A strong SOS *E. coli* promoter fused to a *lux* gene resulted in the development of a construct which served in a dose-dependent detection of 6 genotoxic chemicals including mitomycin C, *N*-methyl-*N*-nitro-*N*-nitrosoguanidine, nalidixic acid, dimethylsulfate, hydrogen peroxide, and formaldehyde [36]. An *E. coli* BL21 DE3 (RIL) biosensor strain displayed a specific response and high sensitivity to different aromatic aldehydes. The response was measured by monitoring the fluorescence of a reporter (green fluorescent protein) fused to an alcohol dehydrogenase inducible promoter (*Sso2536adh*) belonging to the archaeon *Sulfolobus solfataricus* [37]. A plasmid-borne transcriptional fusion between the *E. coli* nitrate reductase (*narG*) promoter and the *Photorhabdus luminescens lux* operon was used to generate a modified *E. coli* with a highly bioluminescent phenotype in the presence of nitrate that enabled the detection of nitrate to a level of 5 × 10^−5^ mol L^−1^ (0.3 ppm) [38]. Following the same approach, biosensors for toluene, arsenite and arsenate, and lead have also been generated [39–41].

In addition to environmental testing and analyses, *E. coli*-based biosensors were found to be useful in the food industry as well. *E. coli* derived β-galactosidase, glucose oxidase, and horseradish peroxidase were immobilized on a glassy carbon electrode to generate a biosensor for quantification of lactose in raw milk [42]. Simultaneous determination of various mono- and disaccharides was performed by a sensor array comprised of bacterial mutants of *E. coli* K12 lacking different transport systems for individual carbohydrates [43].

## 4. *E. coli* as a Biosensor for Amino Acid Bioavailability

Amino acids are building monomers in protein synthesis and indicators for protein quality which explains the interest in constructing microbial biosensors for their quantification. Successful whole-cell biosensors for the quantification of threonine, tryptophan, lysine and glutamine have been developed based on *E. coli* auxotrophy for the respective amino acids [44–46]. Wild type *E. coli* can synthesize all amino acids and does not require their supplementation in media. However, auxotrophic mutants that are defective in the biosynthesis of the amino acid of interest grow in a dose-dependent fashion in response to the external concentration of the amino acid. In addition, *E. coli* is a part of the intestinal microflora of most animals and humans with high similarity in the assimilation of amino acids and peptides which is a necessary prerequisite for the bacterium to serve as a representative biosensor microorganism for these compounds [47]. After pre-treating feed ingredients with pronase and peptidase, Erickson *et al.* [48] obtained a correlation of 0.94 between lysine bioavailability determined by using an *E. coli* lysine auxotroph and previously published chick bioassay data. Indeed, in a direct experimental comparison, the *E. coli* biosensor developed by Chalova *et al.* [19] proved to be as accurate as a chick bioassay for quantitation of bioavailable lysine in diverse feed ingredients and mixtures including soybean meal, cotton seed meal, meat and bone meal, chick starter and finisher, and swine starter.

Early efforts for microbial quantification of methionine have also been based on bacterial auxotrophy. Hitchens *et al.* [45] demonstrated that *E. coli* GUC41 could grow on DL-methionine sulfoxide but not on DL-methionine sulfone. The microbiological assay was as accurate as the chemical assay with high correlation coefficients between the two. The microbiological assay values for amino acid content were expressed as percentages of the HPLC values to obtain the bioavailability values. By using *E. coli* ATCC 23798, a methionine auxotroph, Zabala-Díaz *et al.* [49] were able to quantify crystalline methionine added to feed. The feed matrix had negligible influence on the assay and methionine recovery percentages for all supplementation levels ranged from 71 to 80% indicating consistency in the bacterial response to the supplemented methionine. The *E. coli* methionine growth assay has also been miniaturized and adapted to be conducted in microtiter plates where a linear response of the *E. coli* auxotroph to up to 26.8 μM methionine was achieved [50].

In general, this early assay work with *E. coli* methionine auxotrophs supports the feasibility of this approach and potential reliability for routine use. However, there are also limitations with these particular *E. coli* methionine auxotrophs. To the best of our knowledge, *E. coli* methionine auxotrophs used for methionine quantification so far have been generated via chemical mutation [51–53]. This mutation method is a “hit-or-miss” approach that mutates the organism in random locations, followed by a selection of a certain phenotype. As a result, the mutation is not target specific and various non-methionine related genes can be affected. Revertants or compensatory mutations may occur to abolish the desired functionality [54]. In addition, in the case of methionine, the auxotrophic requirements for this amino acid are not specific and can also be satisfied by a variety of compounds including methioninyl peptides, α-hydroxy methionine, *N*-acetylmethionine, and the α-keto analogue α-keto-λ-methiol butyrate [55]. When a chemically generated *E. coli* methionine auxotroph (ATCC 23798) was used, Froelich *et al*. [56] established no differences based on substrate affinities of an *E. coli* methionine auxotroph to methionine and methionine hydroxy analog, respectively. Estimated maximum growth rate of the *E. coli* auxotroph when grown on both substrates was also found to be similar.

Although the *E. coli* methionine auxotroph did not discriminate between methionine and its hydroxyl analog, it appears that both sources are not equally assimilated by animals. While studying the efficacy of both methionine and methionine hydroxyl analog supplementation of pig diets, Shoveller *et al.* [57] established that methionine hydroxyl analog is significantly less bioavailable compared to DL-methionine for protein deposition in growing pigs. Similar observations were made by Feng *et al.* [58] who reported the methionine hydroxyl analog to be 26.8% and 54.4% less effective than methionine for growing pigs with respect to nitrogen retention and plasma urea nitrogen respectively. Therefore, more specific mutagenesis that targets specific gene(s) without altering other metabolic pathways would be a more desirable approach to generate a microbial biosensor for discriminating and quantifying specific forms of methionine. Detailed knowledge about *E. coli*’s genomics and more specifically, the genes involved in methionine biosynthesis and transportation is a prerequisite to accomplish such a goal and is the focus of the discussion in the following sections.

## 5. Biosynthesis of Methionine in *E. coli*

Methionine’s carbon skeleton is initially derived from aspartate. The intermediates of this pathway, aspartyl semialdehyde and homoserine, are also used in the synthesis of lysine and threonine. Serine and cysteine are metabolically related to the methionine pathway: serine being the precursor in the synthesis of folate, which is the methyl donor for the synthesis of methionine and cysteine from the precursor of cystathionine, which is intermediate in methionine synthesis [59]. Methionine biosynthesis results from the coupling of homocysteine and a methyl group, but can be accomplished via two distinct pathways [55,60]. The *E. coli* K-12 methionine biosynthesis pathway http://biocyc.org/ECOLI/organism-summary?object=ECOLI&detail-level=3 has been schematically presented by EcoCys [61], a member of BioCys database collection (http://biocyc.org/publications.shtml), and is available via SRI International Pathway Tools, version 13.5 [62]. A summary of the consecutive reactions, participating genes and respective products is given in Table 1. The nonfolate branch of the methionine pathway includes *metA, metB, metC, metH, and metK* and the folate branch is comprised of *metF* and *metE* which are all negatively controlled by the *metJ* repressor system [73]. The final methyl transfer is catalyzed by either a B_12_-dependent methyltransferase (*metH* gene product) or a non-B_12_-methyl transferase (*metE* product). The metabolic intermediate, 5-methyltetrahydrafolate, encoded by the *metF*, provides the methyl group for both enzymes to attach. This is a convergence point through which the cells are able to balance the requirement for protein synthesis, methylation reactions, and nucleic acid synthesis on several levels and to regulate the pathway flow of methyl units [73]. Once methionine is formed, it is metabolized to S-adenosylmethionine (AdoMet) in the presence of ATP and AdoMet synthetase, a *metK* gene product [74–76].

The regulation of the methionine biosynthetic pathway consists of positive and negative feedback mechanisms depending on methionine availability and B_12_. For example, MetE biosynthesis is autoregulated via a negative feedback loop. It can also function as an antagonist of the *metR* gene product, by either interfering directly in the activation mechanism or by repressing *metR* expression [73,77,78]. Alternatively, the activation depends not only on the presence of a functional MetR but also on a coactivator. A functional *metF* gene is required for vitamin B_12_- mediated repression of *metE* gene, and 5-methyltetrahydrofolate may be involved in a negative feedback repression. Inactivation of the *metE* gene allows for accumulation of the methionine intermediates O-succinylhomoserine, cystathionine, homocysteine, and 5-methyltetrahydrafolate [60,75].

The first unique step in bacterial methionine biosynthesis involves the activation of homoserine, which in *E. coli* is accomplished through a succinylation reaction catalyzed by homoserine transsuccinylase (HTS). The activity of this enzyme is closely regulated *in vivo* and therefore represents a critical control point for cell growth and viability [79]. Born and Blanchard [80] cloned homoserine transsuccinylase from *E. coli* and demonstrated that the enzyme generates a complex with the succinyl group of succinyl-CoA before transferring it to homoserine to form the final product, O-succinylhomoserine. The enzyme can be inhibited by iodoacetamide in a pH-dependent manner which suggests the presence of cysteine in the active site that forms a succinyl-cysteine intermediate during enzymatic turnover. In *E. coli*, HTS is not only a key regulator of methionine biosynthesis but also of the bacterial growth at elevated temperatures [81]. *E. coli* growth is impaired at temperatures above 44 °C due to the instability of HTS. According to Biran *et al*. [79], the instability of the protein is determined by the amino-terminal part of the protein, and its removal or substitution by the N-terminal part of beta-galactosidase confers stability. Mordukhova *et al*. [82] reported that two amino acids in the enzyme, namely isoleucine 229 and asparagine 267, are responsible for HTS instability and their substitution leads to stabilization of HTS molecule and improved bacterial growth at elevated temperature. MetA is controlled by the expression of *metJ* [83].

MetE, a zinc-containing monomer, transfers the methyl group of N^5^-methyl-tetrahydrofolic to the thiolate group of homocysteine [68,75,78]. Several mechanisms for repression of *metE* exist. The interaction between methionine as S-adenosylmethionine with MetJ leads to a corepression of *metE* through a negative feedback loop [75]. The absence of MetR can also repress *metE*. MetR and MetE are of similar size and exist relatively in the same location but result from transcription in opposite directions [77]. MetR is a transactivator of both *metE* and *metH* gene [75,77,78]. Homocysteine coactivates both the expression of the *metR* gene and the MetR stimulation of *metE* expression.

The vitamin B_12_ -mediated repression of the *metE* gene in *E. coli* requires the B_12_-dependent transmethylase, a product of the *metH* gene. It has been proposed that the MetH-B_12_ holoenzyme complex is involved directly in the repression mechanism [55,73,75]. According to Wu *et al*. [84], however, B_12_-mediated repression of the *metE* gene derives primarily from a loss of MetR-mediated activation due to depletion of the coactivator homocysteine, rather than a direct repression by the MetH-B_12_ holoenzyme. MetH has a higher constant of Michaelis-Menten (K_m_) than MetE, which is compensated by the very strong expression of the *metE* gene [68]. N^5^-methyl-H_4_-folate transfers a methyl unit to the MetH holoenzyme where it is subsequently attached to homocysteine [55,68,75,78]. The cobalamin-independent methyltransferase (MetE) shares no similarity with the sequence of the cobalamin-dependent protein (MetH), suggesting that the two have arisen by convergent evolution [85].

The *metF* gene codes for N^5^-methyl-H_4_-folate and regulates *metE* in an indirect way. N^5^-methyl-H_4_-folate is required for the transfer of a methyl group to the B_12_ within the MetH holoenzyme forming a methyl-B_12_ enzyme; the catalytically active methylated form of the MetH protein regulates *metE* expression [55,75,77,78]. Regulation by MetJ may occur more readily because of the existence of 5 *met* boxes in *metF*’s promotor region making it more sensitive than the other *met* genes to small increases of AdoMet that might occur in B_12_ grown cells [75].

YagD is a third methionine synthase in *E. coli*. YagD is a zinc-dependent methyltransferase with a catalytic mechanism similar to MetH and synthesizes methionine from S-methylmethionine or S-adenosylmethionine and homocysteine. YagD does not contribute to the utilization of methionine sulfoxide as methionine sulfoxide is converted to methionine via reduction. YagD is subject to regulation by the MetJ-S-adenosyl-methionine system [68].

All *met* genes are regulated by MetJ. MetJ protein binds to a specific DNA region, met box, which is present in all *met* genes except *metH*. The met box region is a sequence with dyad symmetry (TGAA . . . TTCA) and produces a helical region containing four leucine residues seven amino acids apart. This motif is called a leucine zipper and has been proposed to play a role in protein dimerization that is required for DNA bindings. MetJ can bind to this region and prevent the transcription of most of the *met* genes (*metA, metBL, metC, metF, metJ, metR,* and *metE*) [55,59,71,74]. The interaction of the MetJ protein with the *met* operator region is markedly enhanced by the presence of AdoMet.

## 6. Bacterial Transport of Methionine

Although *E. coli* prototroph cells are capable of synthesizing methionine *de novo,* they can also acquire external methionine or methionine analogs to satisfy cellular needs for either methionine or sulfur which reflects the high flexibility of the organism under a wide range of environmental conditions. The activity of methionine transport systems in *E. coli* is influenced by the concentrations of both external and internal methionine pools [86]. Cells with increased internal methionine pool or pre-exposed to excess of external methionine exhibit decreased rates of methionine uptake. Conversely, starvation for methionine in a methionine auxotroph can increase the rate of external methionine transport [86].

At least two transport systems for methionine exist in *E. coli*. The high affinity transport system (*metD*) has a K_m_ of approximately 10^−7^ M and is responsible for the uptake of l- and d- methionine isomers [87]. MetD is an ABC transporter with Abc the ATPase, YaeE the permease, and YaeC the likely substrate binding protein. The expression of these genes is regulated by l-methionine and MetJ, the common repressor of the methionine regulon. Interestingly, l-methionine inhibits the uptake of d-methionine; however, d-methionine does little to affect the uptake of the l-isomer [88]. By performing competition experiments Kadner [88] established that MetD possesses a distinct substrate-binding site for each stereoisomer. The second system (*metP*) is a low affinity system with a K_m_ of approximately 40 μM and can transport l-methionine but not the d-isomer [88,89]. By using various deletion mutants, Merlin *et al*. [87] observed that only mutants with active MetD were able to grow on d-methionine.

Methionine can be transported across a concentration gradient (a temperature sensitive uptake process) with the assistance of MetD [90]. The accumulation against a concentration gradient and the temperature influence of the uptake indicates that methionine enters bacterial cells through active transport, which is an energy-dependent process. When starved of methionine, the rate of uptake of methionine is faster than those grown with methionine [91]. Both systems are regulated by the level of the internal methionine pool of the bacterium and differ in affinity by a factor of at least 400-fold [86,88,91]. In *E. coli*, the methionine, transported into the cells, accumulates in the form of AdoMet rather than as free methionine.

Active transport can be completely eliminated in the presence of glucose with the presence of azide and fluoride [86,91]. In the absence of glucose, cells could still accumulate methionine less efficiently. The methionine analogs that inhibit uptake required the −S (or Se)-CHS group [91]. The initial rate of uptake of l-methionine was poorly affected by the addition of α-keto-λ-methiol-butyrate, d-methionine, or methionine sulfoxide when they were added simultaneously with the substrate. However, methionine transport was reduced in cells exposed to analogs and methionine variations prior to the addition of a substrate [86].

## 7. Genetic Strategies for Construction of *E. Coli* Mutants for Methionine Bioassays

### 7.1. General Strategies

Understanding methionine biosynthesis and transportation in *E. coli* is a prerequisite for constructing accurate and specific microbial biosensors for methionine estimation. An *E. coli* microbial bioassay approach for methionine quantification necessitates using an auxotrophic strain for methionine which is incapable of biosynthesizing this amino acid on its own. As discussed in the previous sections, mutants, currently in use for the purpose of methionine quantification, have been developed and isolated after exposure to a chemical mutagen. However, the disadvantage of the imprecise nature of chemical mutagenesis requires other approaches for generation of mutants that are more specific and efficient.

To avoid the hit and miss nature of broad spectrum mutagenesis approaches such as those involving addition of chemical mutagens requires a strategy that targets a specific site on the genome without alteration of the remainder of the genome. Such approaches are more likely to result in phenotypes that are exclusively linked to a specific genetic modification rather than the collective accumulation of several mutations some of which may not be related to the gene(s) of interest. The problem with multiple mutations is not only the risk of unpredictable reversion of the phenotype of interest but a less robust mutant that does not grow as well under the selective conditions required for a particular assay. In the past few years, genetic tools have been developed that harness the utility of biological systems such as transposons that can more directly interact with the bacterial chromosome at specific sites.

Transposons are versatile tools for genetic manipulation and analysis. These are DNA sequences that can be mobilized into bacterial chromosome by a recombination process that is catalyzed by an enzyme, called transposase. In contrast to chemical mutagenesis, insertion of a transposon in the bacterial genome causes complete disruption of the gene of interest and results in non-leaky phenotypes that are specifically linked to the mutated gene [92]. This approach is particularly useful where the function of all the genes in the bacterial genome is not known or the biosynthetic pathway of the analyte of interest is complicated or bypassed. Transposon engineering was used by McAdam *et al*. [93] to mutate *Mycobacterium bovis* BCG, a member of the slow-growing *M. tuberculosis* complex. Two auxotrophs for leucine and one for methionine were isolated from the library of transposon insertions and used to study the functionality of the respective genes. The random insertion of transposon Tn4560 into *Streptomyces tendae* ATCC 31160 resulted in identification of six genes involved in the biosynthesis of nikkornycin, a nucleoside-peptide chitin synthase inhibitor [94]. The scope of application of transposon mutagenesis techniques was increased by Kwon and Ricke [95] and Kwon *et al*. [96] when they developed an approach for the identification of transposon location in the bacterial genome based on the amplification of transposon flanking regions using polymerase chain reaction (PCR).

Deletion of specific gene(s) which abolishes the biosynthetic capability of the bacteria for certain amino acids is an alternative approach to transposon mutagenesis. This technique is applicable when the sequence of the gene to be deleted is known. Four individual (Δ*glnA1*, Δ*glnA2*, Δ*glnA3*, and Δ*glnA4*) and one triple mutant (Δ*glnA1EA2*) of *Mycobacterium tuberculosis* were generated by deletion to investigate the roles of glutamine synthetase enzymes in the nitrogen metabolism of this specific bacterium [97]. Tryptophan auxotrophy in *Leptospira meyeri* was achieved by deletion of the tryptophan biosynthetic gene *trpE* via homologous recombination [98]. Li and Ricke [99] were able to completely delete *lysA* in *E. coli* K12 by using a linear DNA which contained at both ends 50 bp sequences homologous to upstream and downstream sequences of *lysA*. The *lysA* encoded for diaminopimelic acid decarboxylase and is a key enzyme in lysine biosynthetic pathway in *E. coli* K12 [99]. The recombinant strain behaved as an auxotroph for lysine and was not able to grow in minimal medium without lysine supplementation. The *E. coli* K12 Δ *lysA* growth response to increasing concentrations of lysine was found to be linear, which is a must for the purpose of lysine quantification in feed-derived proteins. In fact, after being converted into a fluorescent biosensor, the strain was successfully used to quantitatively assess lysine in feed ingredients and complete diets [19].

### 7.2. Generating specific *E. coli* Methionine Auxotrophs

Identifying a specific gene from the biosynthetic pathway of methionine in *E. coli* in which a deletion could result in methionine auxotroph phenotype is not straightforward. Due to the versatility in methionine biosynthetic pathway, only mutations in certain genes in the *met* regulon would result in auxotrophy for methionine and not for any of the pathway intermediates or precursors. For example, *metL* mutants can grow on homoserine while *metBL* mutants can propagate on both cystathionine and homoserine [101]. While studying mutations that influenced the methionine biosynthetic pathway, Mulligan *et al*. [55] observed that deficiencies in *metA* and *metB* resulted in growth requirements for homosysteine and cystathionine, and a mutation in *metE* was overcome by B_12_ supplementation. Insertions in *metL* and *metH* also did not result in methionine auxotrophy. In the same study, *metF* was the only gene which sufficiently abolished biosynthesis functions to ensure a requirement for external methionine for bacterial growth. In *Streptomyces lividans*, the disruption of a gene encoding for 5,10-methylenetetrahydrofolate reductase which was found to be highly homologous to *E. coli* MetF, also resulted in methionine auxotrophy [102].

In contrast to other amino acid mutants of *E. coli* which require the l-form for growth, *cys* and *met* mutants are capable of using either isomer of cysteine or methionine [103]. d-Methionine is not bioactive and cannot be directly incorporated into protein biosynthesis. Therefore, the utilization of d-methionine for l-methionine is justifiable only if the d-form of this amino acid is ultimately transformed into l-methionine. According to Cooper [104,105], conversion of d- to l-methionine in *E. coli* is possible and occurs via oxidative deamination and subsequent transamination of the keto-methionine product of the former reaction. By using ultraviolet irradiation, Cooper [105] was able to generate a mutant incapable of growing on d-methionine. The locus of the d-methionine utilization was mapped at approximately 2 min away from *lac* region toward threonine and leucine. Therefore, to make an *E. coli* biosensor specific for growth on l-methionine would require an addition to disabling the l-methionine biosynthesis genes as well as the genes responsible for conversion of other forms such as d-methionine transformation to l-methionine. Another approach may be possible now that the structure and allosteric regulation of the high-affinity *E. coli* methionine ABC transporter is better understood [106] and manipulation of methionine transport may offer a more precise targeting of the relationship between intracellular l-methionine transport and external concentration of different forms of methionine.

## 8. Detection Modes for Methionine Microbial Biosensors

As analytical tools, microbial biosensors are genetically engineered to produce a measurable signal in response to the compound of interest. These signals include but are not limited to light emission, reflection, fluorescence, or absorption. Although their function is based on different principles, a common feature is the proportionality between the intensity of the signal and the concentration of target analyte [107]. The choice of an appropriate detection system is an important point since each detection mode possesses advantages and disadvantages which are summarized in Table 2. Several detection methods for detecting microbial responses exist for potential implementation in respect to the microbiological assay for methionine quantification.

### 8.1. Optical Density

Measuring optical density (OD) is a common approach to monitor bacterial growth and is thoroughly reviewed by Kavanagh in [111]. The readings provided by the spectrophotometer correlate directly with the concentration of bacteria in the test media. A non-inoculated tube with media is used to calibrate the spectrophotometer as a representative blank or “zero” value. A nutrient medium is inoculated with the *E. coli* bacterial suspension, incubated at 37°C, and the growth response of the test organism is measured hourly. Over time the turbidity/cell number is calculated and ultimately plotted to determine a linear response. The optical density values that constitute the linear slope gradually increase as the concentration of test nutrient increases.

The theoretical aspects of photometry have been extensively described elsewhere [111]. Although OD measurements require minimal technical effort and are relatively inexpensive there are several drawbacks for their use in quantifying nutrients from complex oranic matrices such as animal feeds and feed ingredients. To prevent any potential alternations of methionine availability, autoclaving or heat treatment of feed samples should be avoided. Therefore, the primary problem is the contribution of nonspecific microflora growth that results in OD increases, not corresponding to different concentrations of the nutrient being quantified by the assay organism. Erickson *et al*. [112] were able to overcome this with the use of an antibiotic-based selective media which exclusively supported the growth of the *E. coli* lysine bioassay organism. Froelich *et al*. [113] tested a medium containing a cocktail of antibiotics and antifungal agents and demonstrated that they did not alter growth kinetics of a methionine *E. coli* auxotroph in response to various methionine concentrations when compared to growth responses of the same strain of *E. coli* grown without antibiotics. Although the use of antibiotics suppress background microflora sufficiently to allow for short term bioassay measurements eventually background microflora can overcome antibiotic inhibition of growth. Consequently, if detection based on OD alone requires longer assay times, background microflora would need to be eliminated by sterilization of the feed matrix. Sterilization, particularly by thermal processes adds to the uncertainty of the accuracy of the amino acid assay by potentially altering their respective availability and any resulting measurements would no longer reflect the original values of the animal feeds being assayed.

### 8.2. β-galactosidase

The measurement of β-galactosidase expression historically is a well-understood, easily measured and reliable method for examining bacterial genetics and understanding fundamentals of gene regulation [114]. The β-galactosidase enzyme assay has also been used as an indirect method of microbial detection and quantitation and is more sensitive than OD [108]. The *E. coli lac* operon enables the organism to metabolize lactose as a carbon source. This *lac* operon is translated at a constant rate when lactose is present in the media [115]. Therefore, the enzyme concentration of lysed cells can be directly correlated with the total bacterial cell count. β-galactosidase assay was successfully used by Hitchens *et al.* [45] to quantify the bioavailability of cysteine, methionine, threonine, and tryptophan in 17 foods. To overcome the lack of exoproteolytic activities in *E. coli*, the food matrices were enzymatically digested with pronase and further subjected to analysis with the respective auxotrophic bacterial strain. The accuracy of the β-galactosidase assay was evaluated by comparison of the data to the amino acid estimates in the same food-derived proteins obtained by a chemical assay. Spearman rank correlation coefficients for the two methods were significant and found to be as follows: cysteine (0.61), methionine (0.95), threonine (0.64), and tryptophan (0.85). Thus, Hitchens *et al*. [45] and earlier Tuffnell and Payne [108] demonstrated that β-galactosidase biosynthesis correlated to the concentrations of the amino acid needed by the auxotrophic bacterial cells for growth and could be accurately used in the quantification of methionine, tryptophan, and lysine bioavailability. However, the β-galactosidase-based assay requires more steps than an OD assay. It is disruptive and is not appropriate for kinetic studies. More importantly as with OD measurements, there is a risk of nonspecific background microflora contributing to overall β-galactosidase assay response as several organisms possess this enzyme. Therefore, a key requirement for any detection system to be used is that it is sufficiently unique *versus* the typical native microflora already present on the animal feed matrix.

### 8.3. Luminescence

Compared to β-galactosidase, luminescence is a more recent detection approach and has been routinely used in the generation of bacterial biosensors. This method allows the detection of viable cells through a quantum measurement to indirectly enumerate cells. A bioluminescent signal can not only be coupled to bacterial growth response to accurately measure levels of the respective nutrient limiting bacterial growth but is 10- fold more sensitive than OD [46,109,116] and considerably more sensitive than β-galactosidase. While testing the efficiency of both firefly luciferase and β-galactosidase as reporters in developing vaccine virus, Rodrigues *et al*. [117] established that the luciferase assay was 1,000-fold more sensitive than that of β-galactosidase. The limit of detection of luminescence produced by the action of luciferase was found to be approximately one infected cell in a background of a million noninfected cells.

In *E. coli*, bioluminescence does not occur and must be acquired via genetic modifications [118,119]. Luminescence is accomplished by the introduction of the *luxAB* genes via plasmid or chromosomal insertion. Cells are subsequently grown in the test media and a chemical reagent is required to induce the bioluminescent phenotype of the inserted sequence. The production of light lasts only minutes (seconds in flash luminescence) before destabilization of the exogenous reagent. This is a shortcoming of luminescence technology and has led to genetic development of longer lasting luminescence and reagent-less requiring strains. The bioluminescence assay response is measured with a flash luminescence luminometer and requires addition of autoinducer [46,116] and therefore, it is not possible to continuously monitor bacterial cell population increases during exponential growth. In order to quantify luminescence, expensive detection devices must be purchased [46,116]. However, Froelich *et al*. [120] demonstrated with a bioluminescent *E. coli* methionine auxotroph that, although the growth kinetics between the transformed strain and a nonplasmid carrying auxotroph were somewhat different, the OD-based standard curves between the two strains were similar. This indicates that even in the absence of available luminescent detection equipment such strains could still be used in a conventional OD-based assay with the advantage being that they could be used for luminescent based assays when the opportunity for using such equipment is made available.

### 8.4. Fluorescence

A similar assay method to luminescence is fluorescence. One advantage of fluorescence over luminescence is that it is less expensive to detect and is a self-contained assay, requiring no additional reagents [121]. Fluorescence occurs naturally in chemicals that resonate (a carbon chain with alternating single and double bonds) [122]. It also occurs in a protein referred to as Green Fluorescent Protein (GFP) that originally was produced in jellyfish (*Aequorea victoria*), but the DNA encoding sequence has been isolated and incorporated via transgenics (the genetic translocation of genes from one species to another, *i.e.*, placing *gfp* from jellyfish into a eukaryotic strain) [123]. Once incorporated, either through transformation of a gene system on a vector or directly incorporated in DNA of the test organism, the fluorescent protein is concomitantly synthesized with other cell proteins. The assay then requires a spectrofluorometer that can excite the engineered organism’s new protein and detect emitted light at a different wavelength [110,124]. Just as with OD, the value are recorded over time and graphed linearly over time and concentration.

Originally discovered in *Aequorea victoria,* two proteins were found within this jellyfish that had luminescent/fluorescent capabilities. The first was aequorin that emitted blue light with the presence of Ca^++^(luminescence). The second was the green fluorescent protein which when excited could be detected on a fluorometer (fluorescence) [125]. Tsien [122] described the general molecular weight of most GFP forms to be approximately 27 kDa. An advantage of the use of *gfp* as a reporter gene is its structural stability. The eleven beta strands surround and protect the chromophore that is positioned near the geometric center of a “beta can”, which protects the chromophore from temperature, acid, and oxidation. Its normal excitation peak is at 395nm with a minor peak at 475nm and emission peaks at 508 nm [122]. It does not require any additional substrates or reagents to fluoresce, and thus sample perturbation and destruction are avoided [126]. Froelich *et al*. [127] successfully transformed an *E. coli* methionine auxotroph with a plasmid encoding for a green fluorescent protein and demonstrated that it could be used to quantify methionine in several representative animal feed ingredients. However, some variation between OD—based measurements and fluorescent measurements were noted suggesting some potential interference with fluorescent measurements.

Some artifacts have to be taken into consideration when detecting the GFP chromophore. Media and feeds may contain aromatic and resonating conjugate carbon chains that may also fluoresce. Some of them have emission spectrum overlapping the emission spectrum of GFP (350 to 550 nm). This often leads to low signal to noise ratios, decreased emission intensity and occasionally complete inability to detect the fluorescence emitted by GFP [128]. To correct for this, a simple excitation filter that allows light to pass at wavelengths higher than 350 nm is used in conjunction with an emissions bandpass filter that allows only light with certain wavelength to pass. Heim and Tsien [129] using specific optical filters detected three different forms of GFP simultaneously in samples of viable bacteria. In addition, GFP variants with different emission spectra were created to overcome either the low intensity of the emission signal or the background fluorescence of various compounds. The resulting GFP mutants are characterized with different excitation/emission spectra, brighter fluorescence, higher solubility, and more even distribution throughout the cytoplasm than the wild type [130]. These mutants allow the monitoring of multiple species of bacteria simultaneously in a complex microbial community. However, Patterson *et al*. [131] implied that no single variant was appropriate for all applications but that each of them offers advantages and disadvantages when investigating viable cells.

There are some issues associated with fluorescence assay which must be accounted for such as autofluorescence from matrices that naturally fluoresce. By studying autofluorecence capacity of feed ingredients including soybean meal, cottonseed meal, meat and bone meal, Chalova *et al.* [132] observed that hydrolyzed feed proteins in concentrations up to 0.1 mg/ml did not interfere with the fluorescence of Gfpmut3 [133] which was used as a reporter in an *E. coli* whole cell-based lysine biosensor. Nonhydrolyzed soybean meal and cottonseed meal did not exhibit detectable background fluoorescence up to 2 mg/ml. The same authors demonstrated the advantages of the constructed *gfp*-based biosensor in the quantification of bioavailable lysine in the feed samples when contaminated with *E. coli*.

The second possible problem is light scatter in the fluorometer. By simply diluting the sample to an OD of 0.1 or less, absorption artifacts and secondary inner filter affects can be avoided. This also prevents light scatter because the density of the sample is lower. Other techniques to lower light scatter should be checked with a blank made from media to determine if scatter is occurring. Finally, the existence of possible quenchers such as other fluorophores which may lower or lose quantum yield can be problematic. This can be corrected with the application of several equations depending on the cause [110].

## 9. Conclusions

In conclusion, microbial sensors for methionine quantification in feed and feed ingredients are an alternative to animal assays because they have the advantage of being simpler, more rapid, and cost efficient. Versatile tools in molecular biology combined with current knowledge of *E. coli* genetics favor the generation of appropriate and successful constructs that may serve as methionine biosensors. However, more work needs to be done in understanding the bacterial genome to better target gene(s) that lead to generation of methionine auxotroph exhibiting a single phenotype. The wide variety of available detection modes should facilitate the choice of a reporting system which will contribute to the simplified operation and identification of a biosensor’s emitted signal.

## Figures and Tables

**Table 1.** Summary of genes which participate in methionine biosynthesis and regulation.

**Gene**	**Product**	**Reaction/Function**	**Reference**
**Methionine biosynthesis**			
*metA*	homoserine O-transsuccinylase	L-homoserine + succinyl-CoA <==> O-succinyl-L-homoserine + coenzyme A	[63]
*metB*	Cystathionine ã-synthetase	L-cysteine + O-succinyl-L-homoserine <==> succinate + L-cystathionine + H+	[64]
*metC*	cystathionase	L-cystathionine + H2O <==> pyruvate + ammonia + L-homocysteine + H+	[65]
*metH*	Cobalamin-dependent tetrahydropteroylglutamate methyltransferase	L-homocysteine + 5-methyltetrahydrofolate <==> L-methionine + tetrahydrofolate	[66]
*metE*	Cobalamin-independent tetrahydropteroyltriglutamate methyltransferase	L-homocysteine + 5-methyltetrahydropteroyltri-L-glutamate <=> L-methionine + tetrahydro-pteroyltri-L-glutamate	[67]
*yagD*	homocysteine methyltransferase	L-homocysteine + S-adenosyl-L-methionine <==> L-methionine + S-adenosyl-L-homocysteine + H+	[68]
*metK*	methionine adenosyltransferase	Catalyzes the formation of the sulfonium compound S-adenosylmethionine from methionine	[70]
**Methionine biosynthesis regulation**			
*metR*	DNA-binding transcriptional activator, homocysteine-binding	Transactivate *metA, metE*, and *metH*	[71]
*metJ*	S-adenosylmethionine transcriptional repressor	Represses transcription from associated promoter	[72]

**Table 2.** Detection systems for microbial assays.

**Detection Systems**	**Characteristics**	**Reference**
Optical Density (OD)	Economical Reliable Easy to use	[111]
â-galactosidase	More sensitive than OD Requires more steps	[108]
Luminescence	10X more sensitive than OD Requires aldehyde to initiate luminescence Expensive	[109]
Fluorescence	Same advantages as luminescence Less expensive detection Self contained assay: no reagents added	[110]
